# Characterization of an NDM-5-producing hypervirulent *Klebsiella pneumoniae* sequence type 65 clone from a lung transplant recipient

**DOI:** 10.1080/22221751.2021.1889932

**Published:** 2021-03-05

**Authors:** Jiankang Zhao, Yulin Zhang, Yanyan Fan, Jiajing Han, Zhujia Xiong, Xinmeng Liu, Binbin Li, Binghuai Lu, Bin Cao

**Affiliations:** a Laboratory of Clinical Microbiology and Infectious Diseases, Department of Pulmonary and Critical Care Medicine, Center of Respiratory Medicine, China–Japan Friendship Hospital, National Clinical Research Center for Respiratory Diseases, Beijing, People’s Republic of China; b Institute of Respiratory Medicine, Chinese Academy of Medical Sciences, Peking Union Medical College, Beijing, People’s Republic of China

**Keywords:** ST65, NDM-5, *Klebsiella pneumoniae*, IncX3, hypervirulence

## Abstract

The emergence of New Delhi Metallo-β-lactamase (NDM)-producing *Klebsiella pneumoniae* has aroused critical concern worldwide. Herein, we reported the first emergence of NDM-5-producing *K. pneumoniae* isolates in a 68-year-old lung transplant recipient, who died of septic shock 13 days after surgery. The *K. pneumoniae* strain KP22937 isolated from the bloodstream of the patient was analyzed for phenotypes and genotypes. KP22937 belonged to sequence type (ST) 65 and capsule serotype K2, contained *iucABCDiutA* and *iroBCDN* virulence clusters, showed high virulence to mice, and was therefore considered a hypervirulent *K. pneumoniae*. The *bla*_NDM-5_ gene was located on a genomic island region of the IncX3-type plasmid pNDM22937, which was successfully transferred to *Escherichia coli* EC600 with insignificant fitness costs. The transconjugant demonstrated similar antimicrobial susceptibility and growth kinetics to the recipient *E. coli* EC600. The plasmid pNDM22937 was almost identical to the *bla*_NDM-5_-carrying IncX3 plasmids previously reported in *K. pneumoniae* strains with different ST types and in other species. Our findings raise concerns about the horizontal spread of *bla*_NDM-5_ gene mediated by IncX3 plasmid, where hypervirulent *K. pneumoniae* strains are also involved. Stricter control measures are needed to prevent the dissemination of the novel clone in hospital settings.

New Delhi Metallo-β-lactamase (NDM) producing *Klebsiella pneumoniae* strains are capable of hydrolyzing all β-lactams except monobactam. The *bla*_NDM-5_ gene was first identified in 2011 from an *Escherichia coli* strain EC045 from a patient with a hospitalization history in India [1]. Subsequently, this carbapenemase gene has been detected in *K. pneumoniae*, and has caused sporadic outbreaks worldwide [2]. It has been identified in various ST types of *K. pneumoniae*, among which ST29 *K. pneumoniae* strain SCNJ1 and ST35 *K. pneumoniae* strain RJY9645 have been confirmed to be carbapenem-resistant hypervirulent *K. pneumoniae* (CR-hvKp) [3,4]. The treatment options are limited for severe infection caused by CR-hvKp [4]. Herein, we reported an NDM-5-producing hypervirulent *K. pneumoniae* ST65 strain in a post-transplant patient, which is a serious public health concern.

In June 2019, a 68-year-old lung transplantation recipient experienced a severe postoperative infection. Multiple specimens were sent for pathogen testing on postoperative day 2, 5, 6 and 7, and empirical antibacterial treatment was administered according to the patient's symptoms until the pathogen results were returned (Figure 1A). Aztreonam was immediately administered after receiving the antimicrobial susceptibility test (AST) result, and the patient's temperature and circulation gradually improved. However, the patient developed chest emphysema on postoperative day 10, and bronchoscopy revealed a large amount of pus moss. His condition dramatically deteriorated, and he died of septic shock.

**Figure 1.**
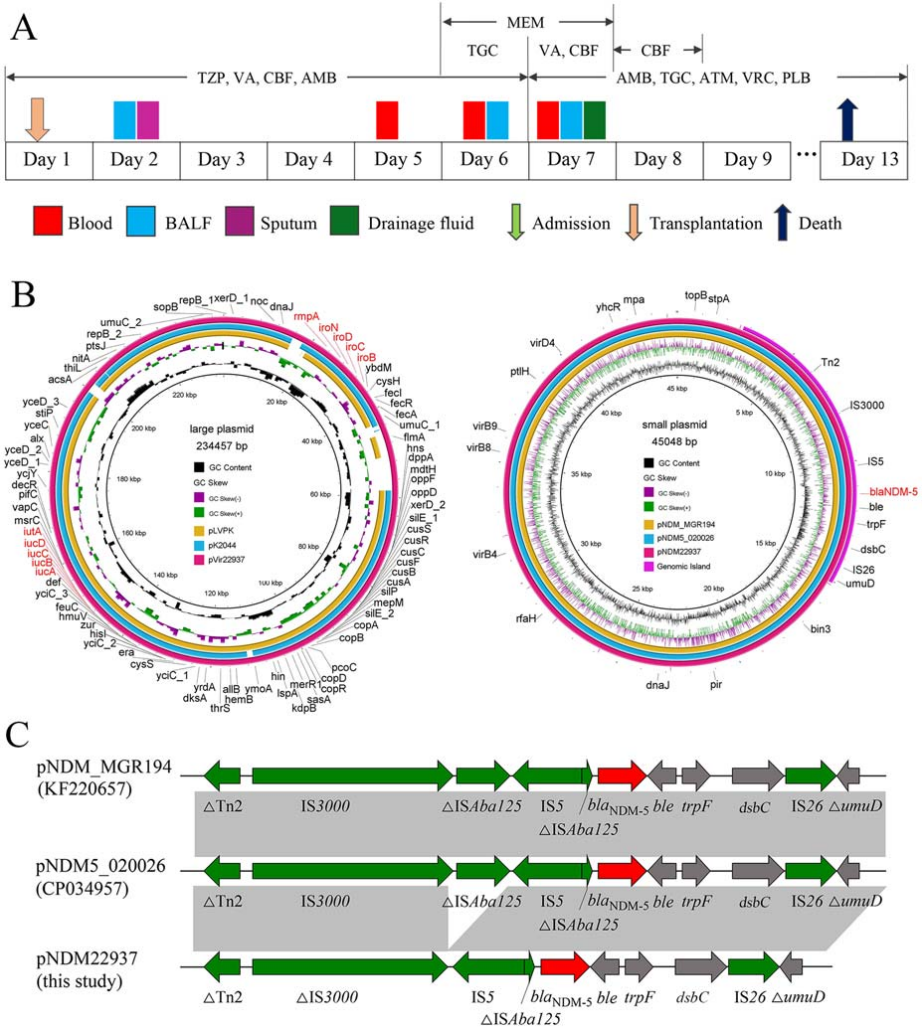
Clinical data of the lung transplantation recipient and genetic features of the two plasmids (pNDM22937 and pVir22937) of *K. pneumoniae* strain KP22937. (A) Time and site of isolation of *K. pneumoniae* from the patient and antimicrobial therapy. TZP, piperacillin/tazobactam; VA, vancomycin; CBF, caspofungin; AMB, amphotericin B; TGC, tigecycline; MEM, meropenem; ATM, aztreonam; VRC, voriconazole; PLB, polymyxin B. (B) Circular plasmid maps of the two plasmids (red colour) and their respective reference plasmids (blue and orange colours). Purple colour region is the genomic island in the plasmid pNDM22937. Red text on the plasmid maps indicates the main virulence determinants and resistance gene of strain KP22937. (C) Genetic contents of *bla*_NDM-5_ gene. ORFs are indicated by arrows. Sequences of shared homology between two plasmids are marked by grey shading.

A total of eight *K. pneumoniae* isolates were recovered from different specimens of the patient (Figure 1A), including blood (*n* = 3), bronchoalveolar lavage fluid (BALF, *n* = 3), sputum (*n* = 1), and drainage fluid (*n* = 1). AST using Vitek-2 system and microdilution broth method showed that these isolates were highly resistant to most antibiotics including carbapenems, cephalosporins, and β-lactam/β-lactamase inhibitors, but were susceptible to aztreonam, aminoglycosides, quinolones, tigecycline and colistin (Table S1). We suspected that these isolates produce Metallo-β-lactamase. Modified carbapenem inactivation method (mCIM) and EDTA-modified carbapenem inactivation method (eCIM) confirmed our suspicion.

We selected the first strain from four different specimens (strain KP22937 from blood; strain KP22866 from BALF; strain KP22877 from sputum; strain KP23025 from drainage fluid; GenBank accession number SAMN17245924-SAMN17245927) for genome sequencing using Illumina HiSeq 2500 sequencing platform, and then strain KP22937 was sequenced using nanopore sequencing method on MinION flow cells to obtain complete plasmid sequences. De novo assembly was conducted using SPAdes Genome Assembler v3.13.1 [5] and Unicycler [6]. Gene prediction was performed using Prokka 1.12 [7]. Genomic islands were predicted using IslandViewer 4 [8]. Antimicrobial resistance genes and plasmid replicon analyses were performed using ResFinder and PlasmidFinder tools via the CGE server (https://cge.cbs.dtu.dk/services/). Virulence genes were identified using the BIGSdb Klebsiella genome database (http://bigsdb.Pasteur.fr/klebsiella/klebsiella.html). Multilocus sequence types (MLST) and K type were determined using Kleborate 0.3.0 (https://github.com/katholt/Kleborate) and Kaptive tool [9], respectively.

The analysis showed that strain KP22937 belonged to ST65 and capsule serotype K2, and carried several hypervirulent determinants such as *iucABCDiutA* and *iroBCDN*, as well as a hypermucoviscous phenotype regulator gene *rmpA*. Mouse lethality assay was performed for assessing the *in vivo* virulence of strain KP22937. The result showed that all eight pathogen-free female BALB/c mice (6–8 weeks and 18–20 g) injected with KP22937 at a concentration of 10^5^ CFU/ml died within 12 h, while mice injected with low-virulence clinical *K. pneumoniae* strain KPZ03 (GenBank accession number SAMN17245928) and PBS survived for over a week (Figure S1). The above results indicated that the ST65 *K. pneumoniae* strain KP22937 that caused infection in the lung transplant recipient was hvKp*,* which was consistent with a previous report [10].

Two plasmids were identified in *K. pneumoniae* strain KP22937. The plasmid pNDM22937, 45048 bp in size, belongs to the IncX3 group and did not harbour any resistance genes other than *bla*_NDM-5_. BLASTN search for GenBank showed that pNDM22937 was nearly identical to *bla*_NDM-5_-carrying plasmids previously reported in *K. pneumonia* of different ST types, and other species. Specifically, pNDM22937 showed 100% identity and coverage with plasmid pNDM5_020026 (CP034957) from *E. coli* SCEC020026, and 100% identity and 97% coverage with the plasmid pNDM_MGR194 (KF220657), which was a typical *bla*_NDM-5_-carrying plasmid recovered from a *K. pneumoniae* isolate in India (Figure 1B) [11]. The genetic environment of *bla*_NDM-5_ gene in pNDM22937 (ΔTn*2*-ΔIS*3000*-IS*5*-ΔIS*Aba125*-*bla*_NDM-5_-*ble-trpF*-*dsbC*-IS*26*-Δ*umuD*) was almost identical to pNDM_MGR194 and pNDM5_020026, except that part of the downstream region of IS*3000* and upstream region of IS*Aba125* were deleted from pKP22937 (Figure 1C). Moreover, we found a genomic island region with 15 open reading frames (ORFs) in the plasmid pNDM22937 (Figure 1B). The first ORF shared 44.6% identity with an integrase from *Serratia sp*, and the last ORF in the genomic island was a twin-arginine translocation (TAT) pathway signal sequence domain protein. The *bla*_NDM-5_ gene and its flanking contents were also present in this region.

Several conjugal transfer genes were also identified in pNDM22937, such as *virB4*, *virB8*, *virB9* and *virD4* (Figure 1B). Conjugation experiment showed that the strain KP22937 transferred the plasmid carrying *bla*_NDM-5_ to the recipient *E. coli* EC600 at a frequency of 10^−5^ (transconjugant/recipient), PCR amplification and sequencing of the transconjugants and S1-PFGE experiment (Figure S2) confirmed successful transfer to the recipient, suggesting that *bla*_NDM-5_ was located on a self-transmissible plasmid, which could mediate dissemination of antibiotic resistance. The recipient *E. coli* EC600 was susceptible to all antibiotics, whereas the transconjugant displayed a similar antibiotic resistance phenotype to the strain KP22937 after obtaining the *bla*_NDM-5_-harbouring plasmid (Table S1). The fitness cost of the IncX3 plasmid harbouring *bla*_NDM-5_ was evaluated through growth kinetics assays. The results showed that the growth of the transconjugant was almost indistinguishable from that of *E. coli* EC600, revealing that the acquisition of IncX3 plasmid did not confer a fitness cost to the host. Accordingly, we hypothesized that the strain that caused postoperative infection in lung transplant patients in this study was produced by obtaining a self-transmissible IncX3 plasmid carrying *bla*_NDM-5_ gene by a hypervirulent ST65 *K. pneumoniae* strain.

In the absence of antibiotic pressure, the presence of resistance genes in plasmids will impose fitness costs on their host [12]. However, a recent *in vitro* study has shown that up to 75.9% (22/29) *Enterobacteriaceae* strains did not produce fitness costs after obtaining the IncX3 plasmid through conjugation test [13], which may facilitate the dissemination of the plasmid. Genomic islands are clusters of genes of probable horizontal origin in bacterial or archaeal genomes. A previous study revealed that genomic islands are a major driver of genome evolution, and they can enhance the fitness of bacteria within a niche [14]. In this study, the *bla*_NDM-5_ gene and its flanking content were located in the genomic island region of plasmid pNDM22937, which may contribute to the lower fitness cost of *K. pneumoniae* strain KP22937. On all accounts, the lower fitness cost may partly explain the rapid dissemination of *bla*_NDM-5_ among *Enterobacteriaceae* strains.

Another plasmid of *K. pneumoniae* KP22937, pVir22937, which was 234457 bp in length, showed 99–100% identity and 91–92% coverage with two classical hypervirulent plasmids pLVPK (NC_005249) and pK2044 (NC_006625) (Figure 1B). Virulence gene clusters *iucABCDiutA* and *iroBCDN*, and *rmpA* gene were located on this plasmid. This plasmid was not self-transmissible, and had no conjugal transfer gene.

In the present study, we reported an NDM-5-producing hypervirulent *K. pneumoniae* ST65 strain. To investigate whether this clone has been previous reported, we retrieved 9157 genomic sequences of *K. pneumoniae* with different assembly levels from the NCBI genome database on July 1, 2020. MLST analysis of these isolates was performed to include only ST65 clone. Eventually, 30 genomic sequences of ST65 *K. pneumoniae* were involved. Analysis of resistance and virulence determinants showed that the *bla*_KPC-2_ gene was present in many ST65 *K. pneumoniae* strains, but the occurrence of *bla*_NDM-5_ gene in ST65 *K. pneumoniae* strain was comparatively rare. This study was the first report that found the *bla*_NDM-5_ gene in a clinical ST65 *K. pneumoniae* strain (Figure S3).

In summary, this study reported a fatal infection in a post-transplant patient caused by an NDM-5-producing hypervirulent *K. pneumoniae* ST65 clone. The *bla*_NDM-5_ gene was located on a self-transmissible IncX3 plasmid, which could be transferred to *E. coli* without significant fitness costs. The current finding raises concerns about horizontal spread of *bla*_NDM-5_ gene mediated by IncX3 plasmid, where hypervirulent *K. pneumonia* strains are also involved.

## Supplementary Material

Figure_S3-heatmap.jpg

Figure_S2-S1-PFGE.jpg

Figure_S1-survival_curve.jpg

Table_S1_AST.docx
