# Supplementary figures and images for: Characterization of an NDM-5-producing hypervirulent Klebsiella pneumoniae sequence type 65 clone from a lung transplant recipient
Source: Emerg Microbes Infect. 2021 Mar 5;10(1):396–9. doi: 10.1080/22221751.2021.1889932 (PMC7939562; doi:10.1080/22221751.2021.1889932)

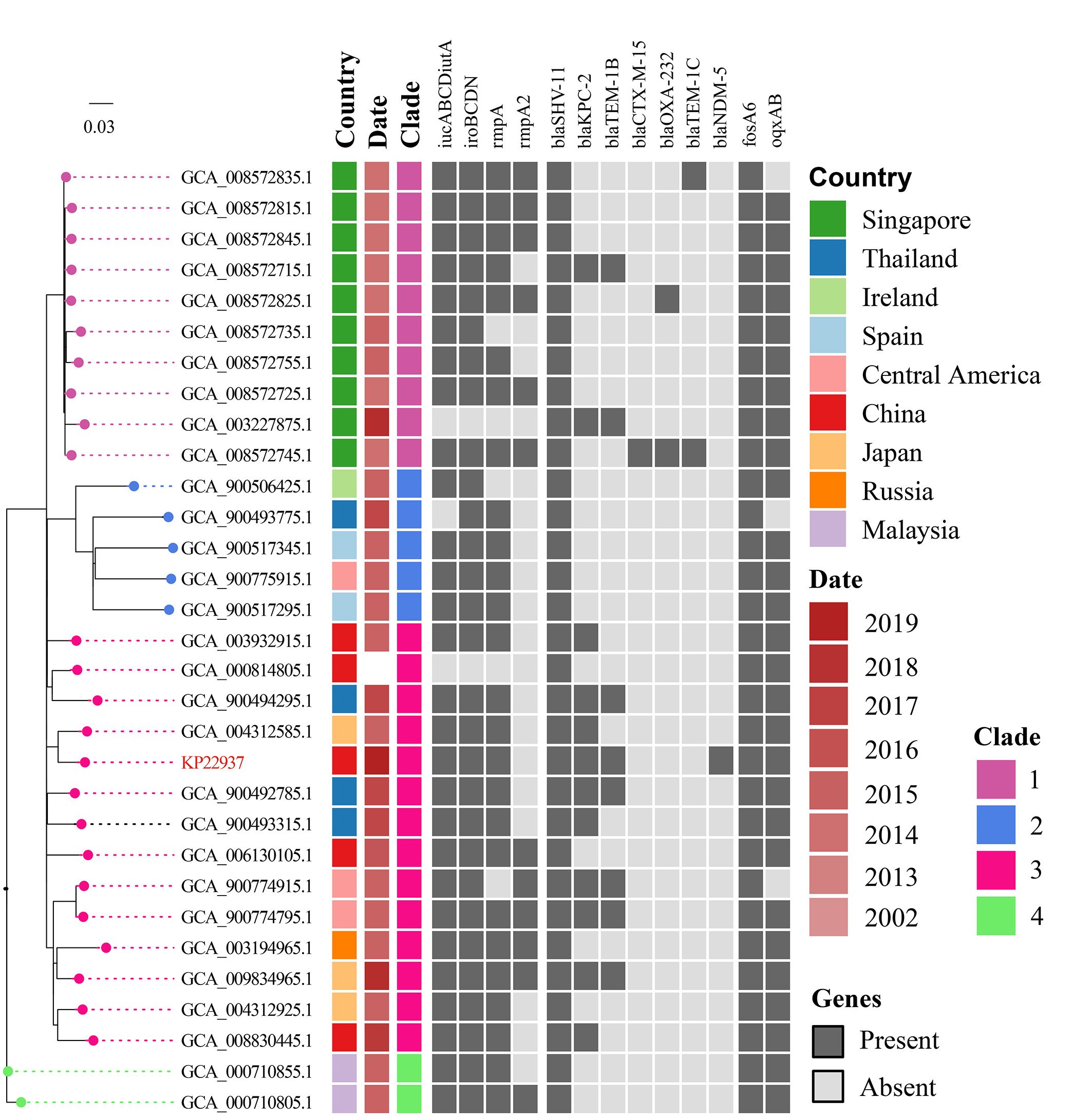

Supplement: Figure_S3-heatmap.jpg [file TEMI_A_1889932_SM4202.jpg]

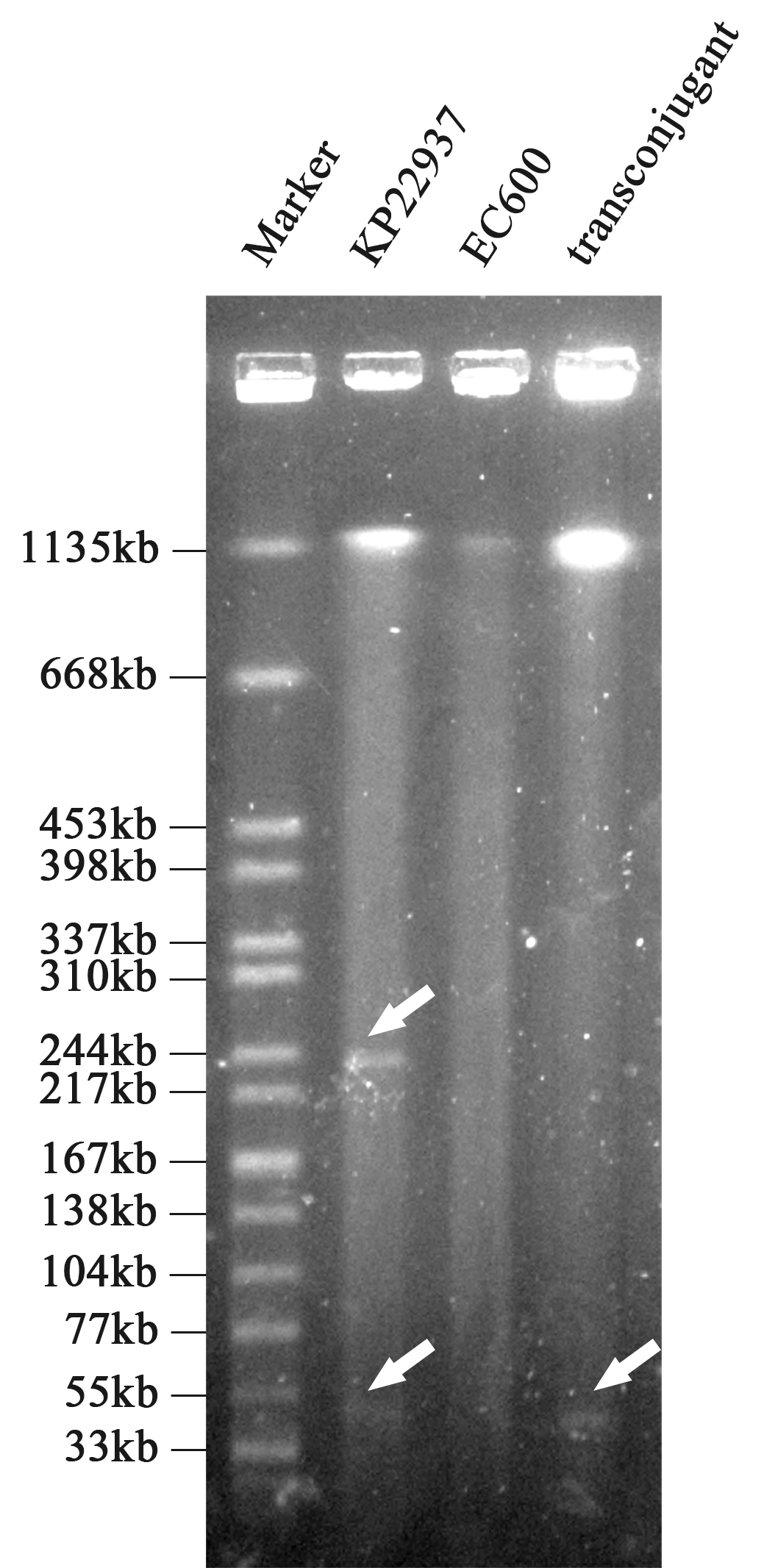

Supplement: Figure_S2-S1-PFGE.jpg [file TEMI_A_1889932_SM4201.jpg]

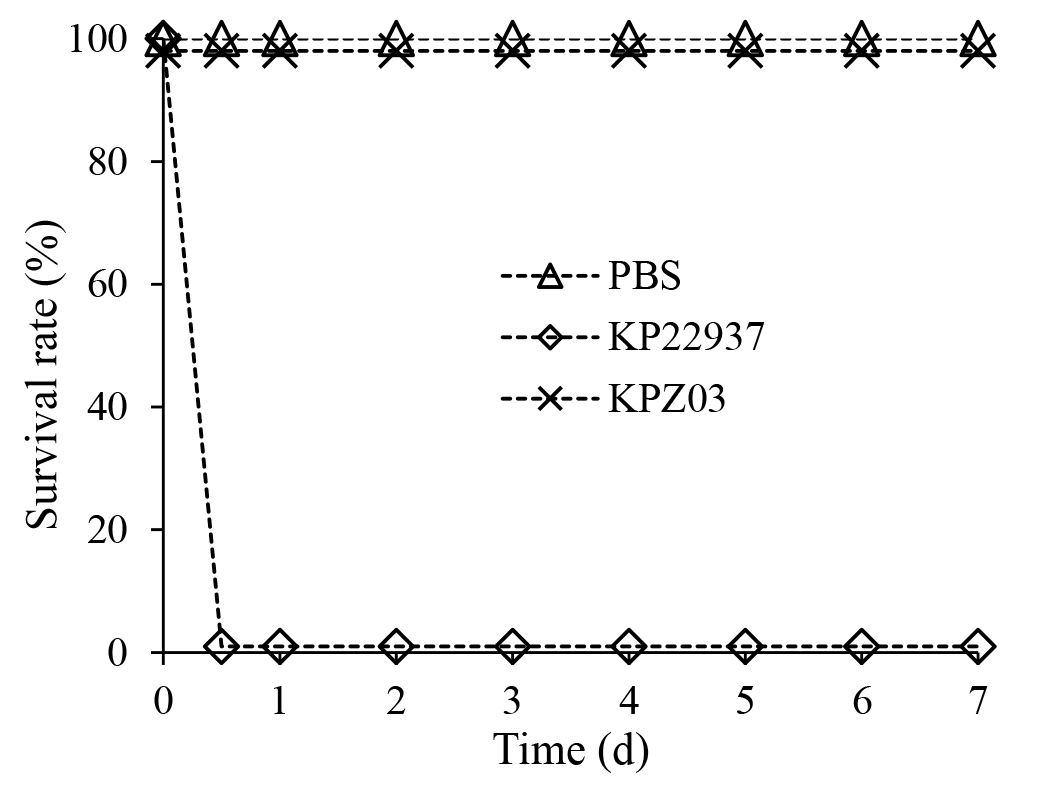

Supplement: Figure_S1-survival_curve.jpg [file TEMI_A_1889932_SM4200.jpg]
