# Supplementary material for: Characterization of an NDM-5-producing hypervirulent Klebsiella pneumoniae sequence type 65 clone from a lung transplant recipient
Source: Emerg Microbes Infect. 2021 Mar 5;10(1):396–9. doi: 10.1080/22221751.2021.1889932 (PMC7939562; doi:10.1080/22221751.2021.1889932)
Supplement: Table_S1_AST.docx [file TEMI_A_1889932_SM4199.docx]

Table S1. Antibiotic resistance characteristics (MICs, mg/L) of 4 clinical *K. pneumoniae* strains and the transconjugant of KP22937 detected using Vitek-2 system.

| Antibiotics | KP22937 | KP22866 | KP22877 | KP23025 | EC600 | Transconjugant |
| --- | --- | --- | --- | --- | --- | --- |
| Amikacin | <=2 | <=2 | <=2 | <=2 | <=2 | <=2 |
| Tobramycin | <=1 | <=1 | <=1 | <=1 | <=1 | <=1 |
| Gentamicin | <=1 | <=1 | <=1 | <=1 | <=1 | <=1 |
| Tigecycline^a^ | 0.5 | 0.5 | 0.5 | 0.5 | 0.25 | 0.25 |
| Cefazolin | >=64 | >=64 | >=64 | >=64 | <=4 | >=64 |
| Cefepime | >=32 | >=32 | >=64 | >=64 | <=1 | 16 |
| Ceftriaxone | >=64 | >=64 | >=64 | >=64 | <=1 | >=64 |
| Ceftazidine | >=64 | >=64 | >=64 | >=64 | <=1 | >=64 |
| SAM | >=32 | >=32 | >=32 | >=32 | 8 | >=32 |
| TZP | >=128 | >=128 | >=128 | >=128 | <=4 | >=128 |
| SCF | >=64 | >=64 | >=64 | >=64 | <=8 | >=64 |
| Aztreonam | <=1 | <=1 | <=1 | <=1 | <=1 | <=1 |
| Imipenem | >=16 | >=16 | >=16 | >=16 | <=0.25 | >=16 |
| Meropenem | >=16 | >=16 | >=16 | >=16 | <=0.25 | >=16 |
| Levofloxacin | <=0.12 | 0.5 | <=0.25 | <=0.25 | 0.5 | 0.5 |
| Ciprofloxacin | <=0.25 | <=0.25 | <=0.25 | <=0.25 | <=0.25 | <=0.25 |
| Colistin | <=0.5 | <=0.5 | <=0.5 | <=0.5 | <=0.5 | <=0.5 |
| SXT | <=20 | <=20 | <=20 | <=20 | <=20 | <=20 |

^a^MIC of tigecycline was detected using microdilution broth method. SAM, Ampicillin/sulbactam; TZP, Piperacillin/tazobactam; SCF, Cefoperazone/sulbactam; SXT, Sulfamethoxazole/trimethoprim.
